# Atypical and incomplete pulmonary hypertrophic osteoarthropathy in the left distal femur: a case report

**DOI:** 10.1186/s12893-020-00959-6

**Published:** 2020-11-23

**Authors:** Gang Xu, Norio Yamamoto, Katsuhiro Hayashi, Akihiko Takeuchi, Shinji Miwa, Kentaro Igarashi, Yuta Taniguchi, Yoshihiro Araki, Hirotaka Yonezawa, Sei Morinaga, Hiroyuki Tsuchiya

**Affiliations:** Department of Orthopaedic Surgery, Kanazawa University School of Medicine, 13-1 Takara-machi, Kanazawa, Ishikawa 920-8641 Japan

**Keywords:** Pulmonary hypertrophic osteoarthropathy, Unilateral symptom, Low-grade osteosarcoma, Benign lesion, Clubbing

## Abstract

**Background:**

Pulmonary hypertrophic osteoarthropathy (PHO) is a rare paraneoplastic syndrome that mainly occurs in patients with lung cancer. Most symptoms occur symmetrically, and unilateral symptoms without clubbing are infrequent. This report presents the case of a rare atypical symptom that was highly suspected of being PHO.

**Case presentation:**

A 77-year-old woman with swelling and severe pain in the left femur for 2 months was referred to our hospital. Radiography revealed a remarkably osteogenic thickening and sclerotic lesion in her distal femur. Preliminary diagnoses of malignant bone tumor and hematological tumor were made based on laboratory test results, radiological examination, and clinical manifestation. A needle biopsy was performed, which ruled out the diagnosis of malignant bone tumors. A low-grade bone tumor was still suspected. After that, en bloc resection was performed, followed by replacement of the femur with a mega-prosthesis. Pathological analysis revealed no malignant tumor cells, and immunohistochemical staining for CDK4 and MDM2 yielded negative results. Meanwhile, pulmonary large cell carcinoma was identified on biopsy. Based on published studies, a diagnosis of atypical PHO was made according to clinical and imaging manifestations.

**Conclusions:**

This is an infrequent case of PHO with unilateral symptoms in the left femur. When clinical manifestations and radiological findings are inconsistent with the pathological results, the possibility of benign lesions with malignant clinical manifestations or imaging features should be carefully considered.

## Background

Hypertrophic osteoarthropathy (HOA) is a rare syndrome that can be divided into two categories: primary (hereditary or idiopathic) and secondary HOA. Pulmonary hypertrophic osteoarthropathy (PHO) can be secondary to HOA, which is a paraneoplastic syndrome. Most patients are complicated by PHO secondary to intrathoracic malignancies, lung or heart diseases. PHO predominantly usually occurs in middle-aged and elderly patients. The incidence of PHO is relatively low, and prior studies have reported incidences of PHO of 0.2–17% in lung cancer patients [1–3].

Clinical symptoms of PHO usually appear symmetrically in the extremity and include clubbing, periostitis, arthritis, periosteal proliferation, and pain and swelling in the joints [4]. Radiological examination and bone scanning are the mainstays of diagnosis. Mainly, bone scintigraphy is an extraordinarily sensitive technique to diagnose PHO, “with the presence of the parallel track sign” that shows symmetrical increasing uptake along the cortex of the diaphysis and metaphysis in long bones [5].

According to prior studies, PHO with unilateral symptoms or without clubbing is relatively uncommon. This report presents a rare atypical symptom that was highly suspected of being PHO accompanied by unilateral pain, periostitis, and swelling. The clinical symptoms and radiological findings was similar to those of malignant tumor. This case will be expected to provide knowledge for the accurate diagnosis of atypical PHO with unilateral symptoms in the extremities.

## Case presentation

**Fig. 1:**
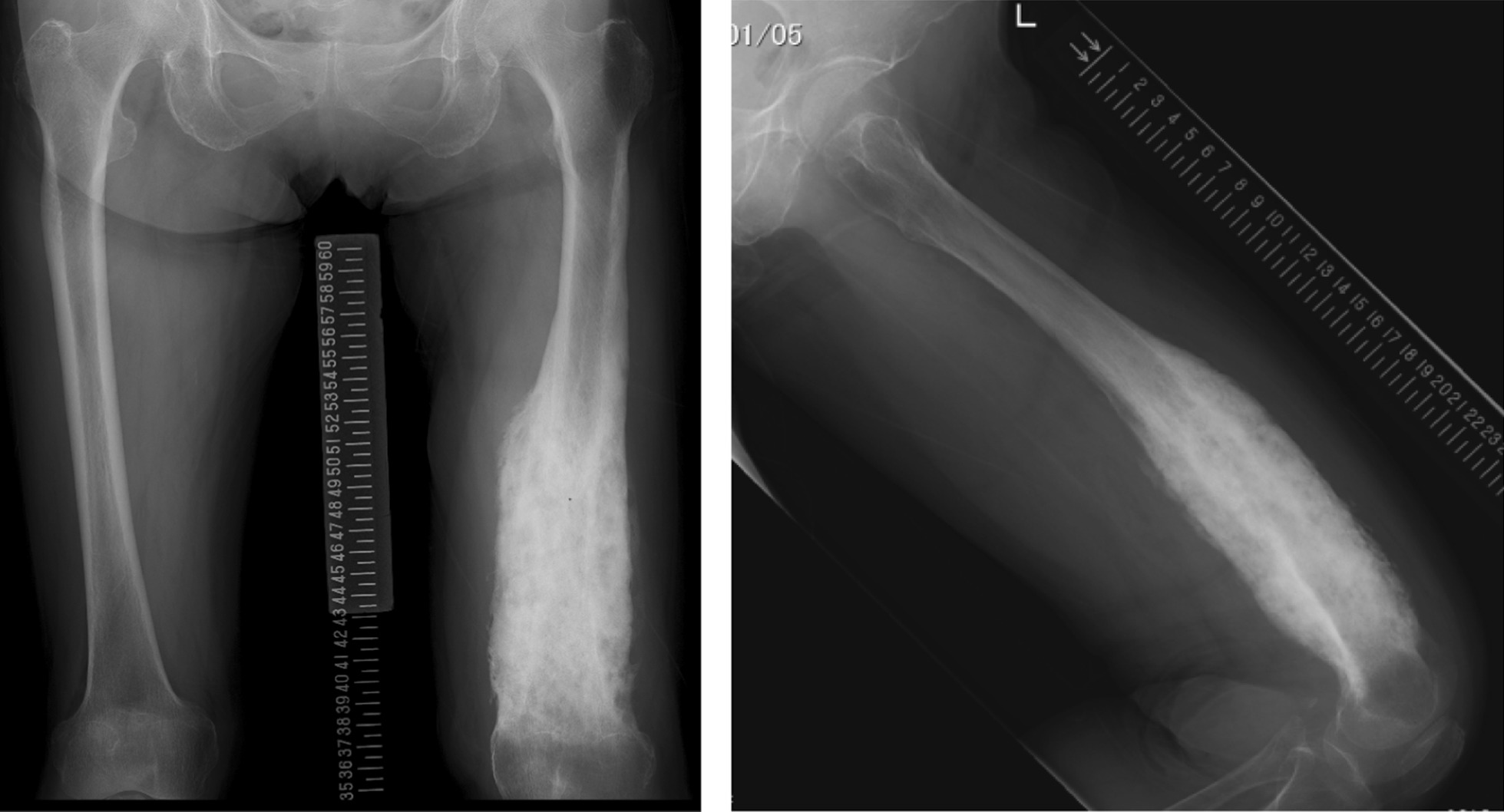
Radiograph of the left femur. Radiograph of the left femur showing an extensively osteogenic, thickened and sclerotic lesion from the mid-diaphysis to the distal femur. A dense, irregular margin was observed in the intramedullary and cortical lesions. The length of the sclerotic lesion is approximately 18 cm

In January 2018, a 77-year-old woman was referred to our hospital with spontaneous pain and swelling of the left thigh. The patient noticed swelling of the left distal thigh, without pain, in 2015. In November 2017, she developed severe pain and consulted doctors at a general hospital. Radiography revealed a remarkably osteogenic thickening and sclerotic lesion in the distal femur (Fig. 1). The preliminary diagnosis of malignant bone tumor and aplastic anemia were made at the hospital before transfer to our hospital. Physical examination showed local tenderness and a hard bony swelling of the femur but no redness or local heat.

Laboratory test results were as follows: white blood cell: 2870/μL, hemoglobin: 8.5 g/dL, blood platelet: 22 × 10^4^/μL, and neutrophil: 2.24 × 10^9^/L. The hematologist suspected aplastic anemia, as the blood cell levels were three-times lower than the typical values and suggested symptomatic treatment.

**Fig. 2:**
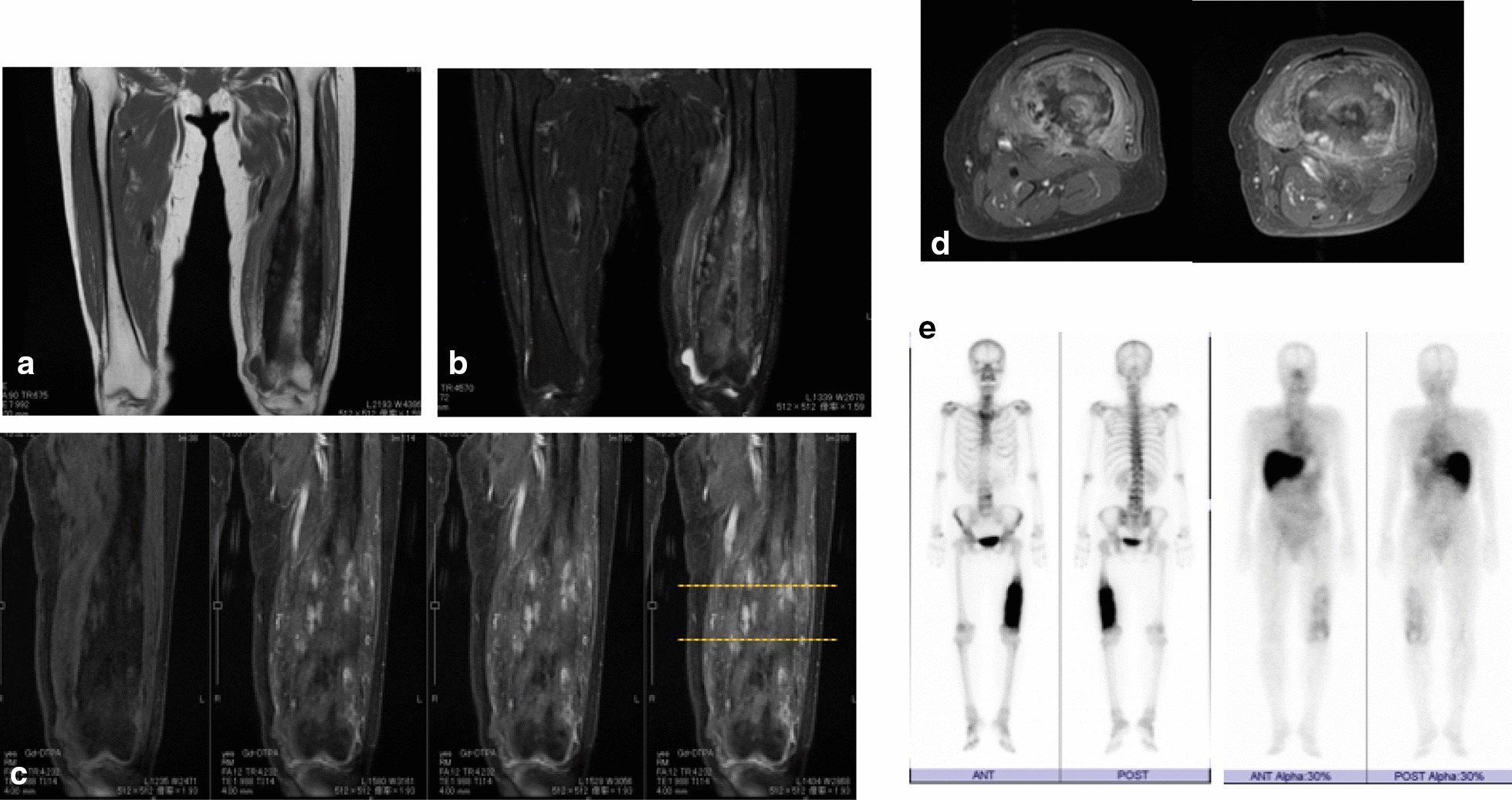
MRI and bone scan of the left femur**.** MRI image of the left femur. **a** T1-weighted image showing an extremely low signal intensity and a heterogeneous lesion in the sclerotic lesion and medullary cavity, respectively. The medullary cavity is narrower than that in the contralateral femur. **b** T2 fat-suppressed image showing heterogeneous signals in the sclerotic lesion and medullary cavity. **c**, **d** Gadolinium contrast MRI images showing partially high signals around the bone and axial image showing heterogeneous signals in the medullary cavity and soft tissue. **e** Bone scans showing significant uptake in the left femur

On magnetic resonance imaging (MRI), the T1-weighted image showed extremely low signals in the sclerotic lesion. The T2 fat-suppressed image showed heterogeneously signals in the sclerotic lesion and medullary cavity. Gadolinium contrast MRI images showed multiple high signals in the soft tissue and bone. The bone scintigraphy revealed significant uptake in the left femur (Fig. 2).

**Fig. 3:**
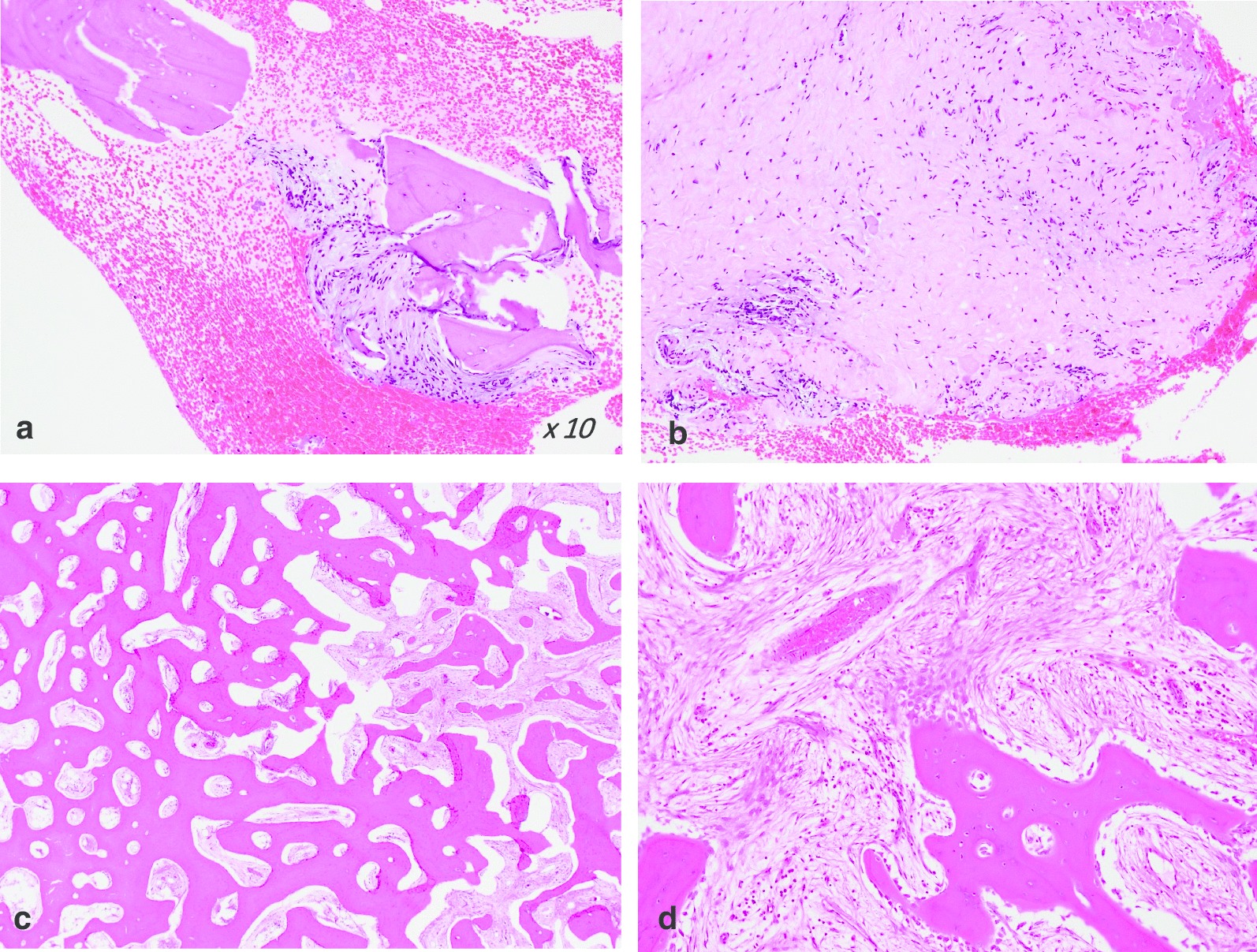
Results of needle biopsy. **a**, **b** Results of the first needle biopsy showing fibrous tissue and granulation tissue around the bone matrix and no malignant cells in the section. **c**, **d** Final histopathological results showing massive new bone formation and osteoblast cells in the area of new bone formation, with no malignant tumor cells

Based on the above findings, the diagnosis of osteosarcoma and hematological malignancy was made. Meanwhile, a needle biopsy was carried out, and histopathological examination showed no malignant tumor cells in the specimen and negative bone marrow biopsy results (Fig. 3a).

**Fig. 4:**
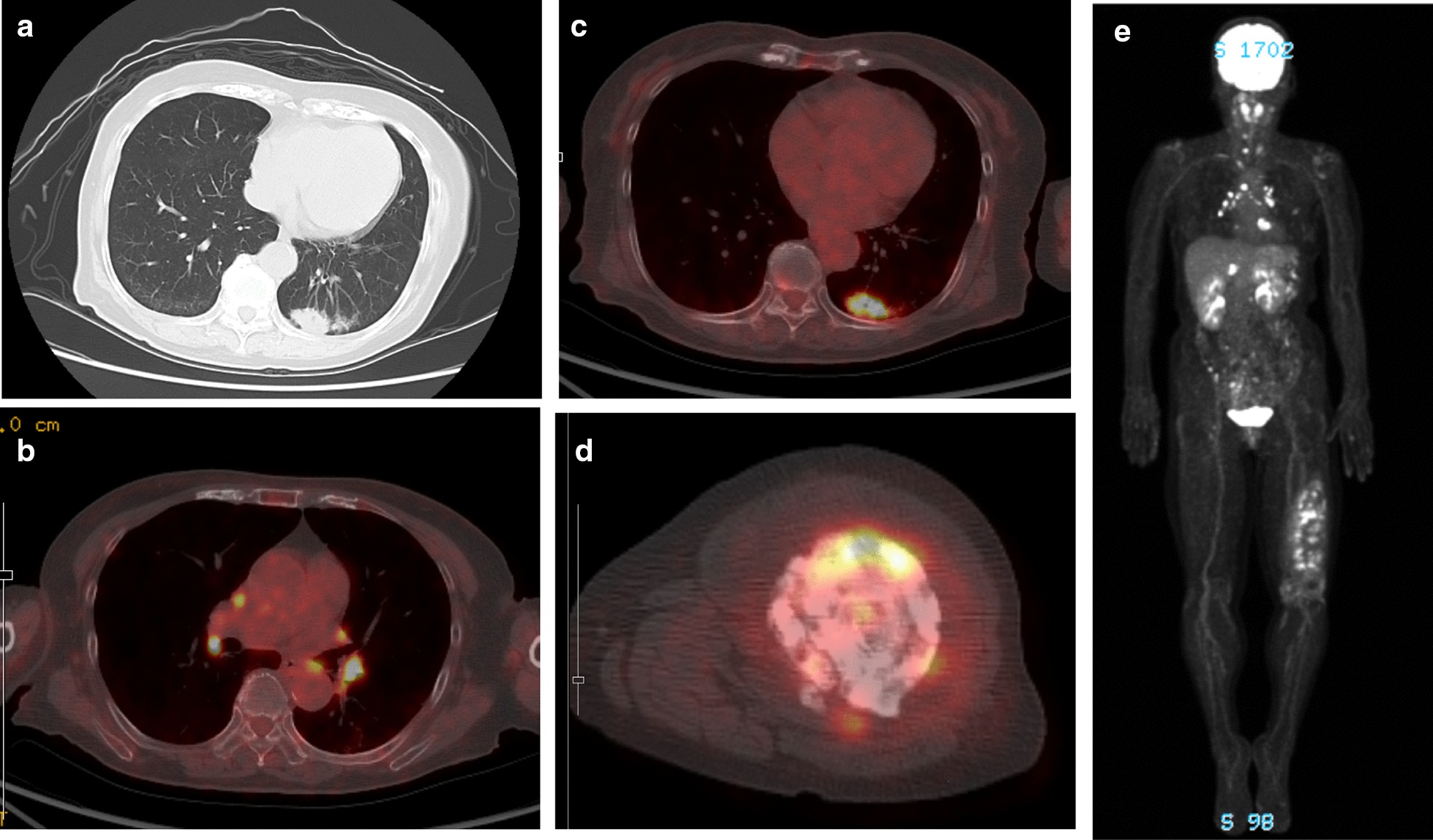
Results of Lung CT scan and 18F-FDG PET-CT scan. **a** Chest computed tomography scan showing a solitary pulmonary nodule in the left lower lobe. **b**–**e** 18F-FDG PET-CT scan showing simultaneous uptake in the lung, lung lymph node, cervical vertebra, thoracic vertebra, and right third rib and partially high uptake in the left distal femur

Thereafter, 18F-fluorodeoxyglucose-positron emission tomography-computed tomography (PET-CT) showed abnormally high accumulation in the lung, lung lymph nodes, cervical vertebra, thoracic vertebra, right third rib and left femur (Fig. 4).

**Fig. 5:**
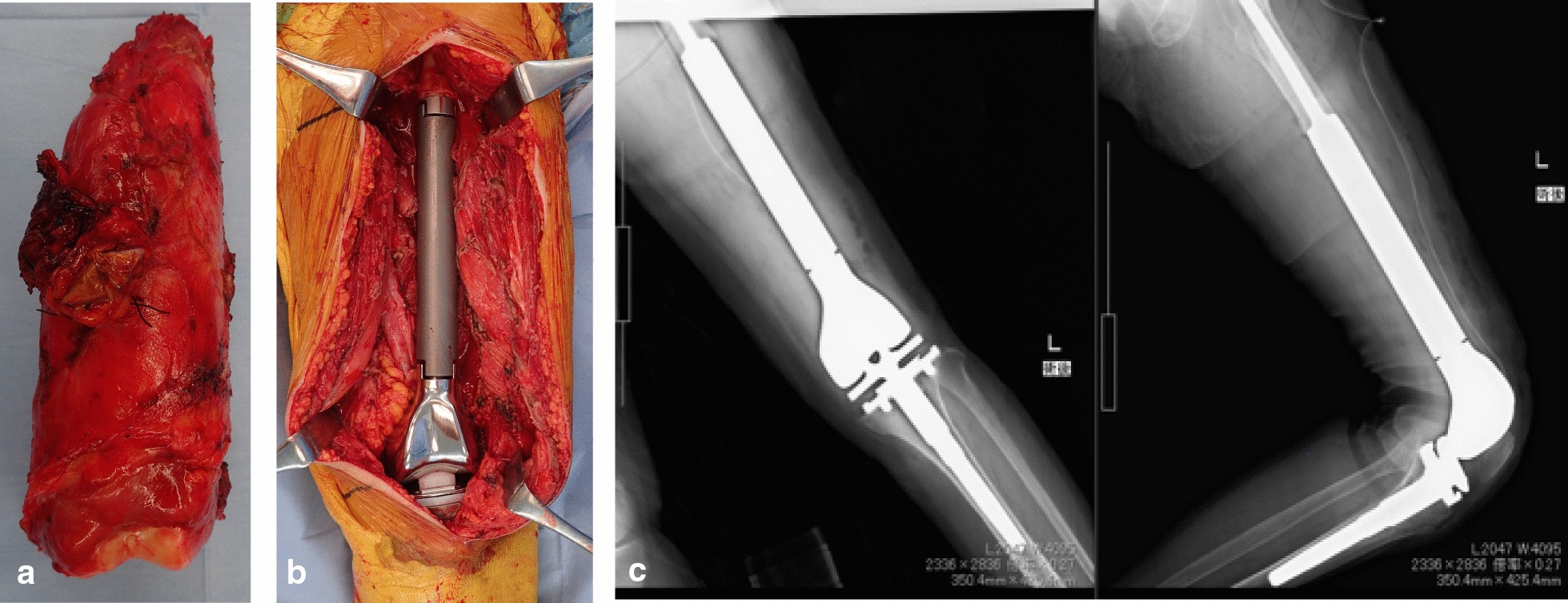
Intraoperative image and postoperative radiograph. **a** Gross specimen of the resected distal femur. **b** Intraoperative image showing mid-diaphysis to distal femur replacement by a mega-prosthesis. **c** Postoperative radiograph showing the femur after replacement with a mega-prosthesis

The clinical manifestations and radiological examination findings were inconsistent with the pathological results. However, a diagnosis of the low-grade malignant bone tumor was still suspected by the multidisciplinary team (MDT), and a treatment plan involving complete excision was done. Because of the older age, poor medical condition and suspicion of a low-grade tumor, neoadjuvant chemotherapy and open biopsy were not performed. En bloc resection was carried out, followed by the replacement of the distal femur with a mega-prosthesis (Fig. 5). The final pathological analysis showed no malignant tumor cells and negative immunohistochemical staining results for CDK4 and MDM2; therefore, ruling out a diagnosis of the low-grade malignant bone tumor (Fig. 3c). Meanwhile, staining for CD3, CD20, CD34, and kappa and lambda light chains were negative and hematological malignancies were ruled out. Considering the PET-CT revealed high uptake in the lung and lung lymph nodes, a CT-guided percutaneous puncture biopsy was done which revealed pulmonary large cell carcinoma, and the patient was eventually transferred to the Respiratory Department.

Due to the lack of support, all malignant tumors were ruled out. Combined with imaging diagnosis such as significant uptake in bone scan and PET-CT and sclerotic lesion in radiography, meanwhile patient was also diagnosed as pulmonary large cell carcinoma, PHO was finally diagnosed.

## Discussion and conclusions

Low-grade osteosarcoma and benign lesions are difficult to distinguish, especially osteoblastic lesions in the distal femur, due to its non-specific radiological findings and clinical manifestation. Since there are no atypical malignant tumor cells in the histological section, it is easy to be misdiagnosed, which leading to a poor outcome. Previous studies have reported that the misdiagnosis rate was close to 50% with a preoperative biopsy [6, 7]. Even specialized pathologists find it challenging to make a diagnosis, requiring multiple biopsies and massive amounts of the specimen. Currently, the expression of MDM2 and CDK4, as sensitive markers on immunohistochemical (IHC) staining is used in the distinction of low-grade osteosarcoma in most cases [8].

Hayashi et al. [9] reported a case of low-grade osteosarcoma in which the pathologists had to perform histological examination four times to confirm the diagnosis—this case was previously diagnosed as fibrous dysplasia.

In our study, the preliminary diagnoses were osteosarcoma and hematological malignancy based on clinical and radiographic characteristics. Subsequently, considering the PET-CT results, a metastatic tumor, osteomyelitis and a low-grade malignant bone tumor were suspected. However, although histopathological examination and bone-marrow biopsy excluded the above-mentioned diseases, the low-grade malignant bone tumor was still highly suspected. Initially, an open biopsy was planned to obtain more specimens to arrive at a definitive diagnosis. However, given the fact that multiple biopsies and extraction of the massive specimen might increase the risk of transmission of tumor cells, as well as, the patient having aplastic anemia and old age, there was a possibility that the patient could not tolerate multiple operations and anesthesia exposure. In addition, considering the patient had severe pain and poor function of the left femur. Therefore, we had to adopt a surgical plan of en-bloc resection of the tumor at the femur and followed by replacement with megaprosthesis.

The resected specimen was then sent for histopathological examination to determine the definitive diagnosis. Unfortunately, the final histopathological analysis revealed massive fibrous tissue, granulation tissue, osteoblasts and no malignant tumor cells. Hence, the diagnosis of low-grade osteosarcoma was ruled out by the results of MDM2 and CDK4 on IHC staining.

Consequently, the pulmonary large cell carcinoma was identified on biopsy. In addition, the pathologist also performed the IHC staining for CD3, CD20, CD34, and kappa and lambda light chains, which yielded negative results, ruling out the diagnosis of hematological malignancies. Therefore, malignant bone tumors, hematological malignancies, low-grade osteosarcoma, and metastatic tumors were all completely excluded. Nonetheless, it was noted that the severe pain and limb function significantly improved after the surgery.

Due to clinical manifestations and radiological examination findings were inconsistent with the pathological result, it is difficult to make a final diagnosis in this case. Gene detection might help make a more accurate diagnosis according to relevant literature [10]. Unfortunately, gene detection is difficult to perform in our hospital. Although we considered a diagnosis of PHO after the first biopsy, we were more inclined to diagnose a low-grade bone tumor as the patient’s symptoms were merely associated with the unilateral extremity, and no clubbing was observed, which are rare findings. After the final histopathological examination, all differential diagnoses could be reasonably excluded, leading to a high suspicion of PHO.

Clinical manifestations of PHO include clubbing, periostitis, arthritis, periosteal proliferation, and pain and swelling in the joints, which appear symmetrically in the extremity [4]. Radiological examination and bone scanning are used for diagnosis. The presence of the “parallel track sign” shows that the symmetrical uptake of the long bone diaphysis and metaphysis cortex are increased. PHO often occurs in patients with non-small cell carcinoma according to published literature [11], which is the same as our result with large cell carcinoma a biopsy. The patient also lacked pulmonary symptoms at the time of the initial presentation. Remarkably, we were unable to identify the sequence of events between the development of lung cancer and femoral symptoms. Nevertheless, paraneoplastic syndrome developed earlier than lung cancer [12]. In addition, Ali et al. reported that PHO without clubbing is an incomplete form of PHO [13]. Likewise, there are a few studies that have reported on unilateral symptoms with PHO [14, 15], and PHO increased fluorodeoxyglucose uptake on PET-CT [16]. Thus, we highly suspected a diagnosis of atypical PHO based on the clinical and radiological manifestations.

The standard treatment for PHO is the removal of the primary lesion. Chemotherapy or radiotherapy are acceptable methods for control of the unresectable primary disease. Administration of adrenocortical hormone, nonsteroidal anti-inflammatory medications, and opioids have been reported to be useful in pain management. However, in this case, even if the diagnosis of PHO was confirmed in the patient before surgery, it would have been difficult to administer chemotherapy or palliative treatments because of the poor condition of the patient [17].

This is a very rare case, and we can't make a definitive diagnosis. Due to the lack of typical symptoms, we combined the symptoms of the patient with the relevant literature. Finally, we had to highly suspect the diagnosis of PHO, although it does not have typical PHO symptoms. However, due to the result of the PET-CT examination, we did not examine some serum tumor markers. In addition, the patient had severe pain and poor function of the left femur, and we do not know if conservative treatment can relieve the symptom. We hope that this case can provide more ideas for the diagnosis of tumors in the future.

In conclusion, we reported a case in which the clinical manifestations and radiological examinations results are inconsistent with the pathological results. We should carefully consider the clinical manifestations with atypical symptoms, radiological examination results, and pathological results between the benign lesion and malignant tumors. We believe that this case report has clinical applicability in making a differential diagnosis between a malignant bone tumor and a benign lesion.

## Data Availability

All data generated or analyzed during this study are included in this published article.

## Abbreviations

HOA: Hypertrophic osteoarthropathy
PHO: Pulmonary hypertrophic osteoarthropathy
MRI: Magnetic resonance imaging
PET-CT: Positron emission tomography-computerized tomography
IHC: Immunohistochemical
MDM2: Murine double minute 2
CDK4: Cyclin-dependent kinase 4
